# The Higher Prevalence of Venous Thromboembolism in the Hungarian Roma Population Could Be Due to Elevated Genetic Risk and Stronger Gene-Environmental Interactions

**DOI:** 10.3389/fcvm.2021.647416

**Published:** 2021-10-26

**Authors:** Shewaye Fituma Natae, Zsigmond Kósa, János Sándor, Mohammed Abdulridha Merzah, Zsuzsanna Bereczky, Péter Pikó, Róza Ádány, Szilvia Fiatal

**Affiliations:** ^1^Department of Public Health and Epidemiology, Faculty of Medicine, University of Debrecen, Debrecen, Hungary; ^2^Doctoral School of Health Sciences, University of Debrecen, Debrecen, Hungary; ^3^Department of Health Methodology and Public Health, Faculty of Health, University of Debrecen, Nyíregyháza, Hungary; ^4^Division of Clinical Laboratory Science, Department of Laboratory Medicine, Faculty of Medicine, University of Debrecen, Debrecen, Hungary; ^5^Magyar Tudományos Akadémia-Debreceni Egyetem (MTA-DE) Public Health Research Group, University of Debrecen, Debrecen, Hungary

**Keywords:** VTE, GxE interactions, ATBp3 mutation, SERPINC1, Roma population, general Hungarian

## Abstract

**Background:** Interactions between genetic and environmental risk factors (GxE) contribute to an increased risk of venous thromboembolism (VTE). Understanding how these factors interact provides insight for the early identification of at-risk groups within a population and creates an opportunity to apply appropriate preventive and curative measures.

**Objective:** To estimate and compare GxE for VTE risk in the general Hungarian and Roma populations.

**Methods:** The study was based on data extracted from a database consisting of results previously obtained from a complex health survey with three pillars (questionnaire-based, physical, and laboratory examinations) involving 406 general Hungarian and 395 Roma subjects. DNA was genotyped for rs121909567 (SERPINC1), rs1799963 (F2), rs2036914 (F11), rs2066865 (FGG), rs6025 (F5), and rs8176719 (ABO) polymorphisms. After allele frequency comparisons, the odds ratio (OR) was calculated for individual SNPs. Furthermore, genetic risk scores (weighted GRS, unweighted GRS) were computed to estimate the joint effect of the genetic factors. Multivariable linear regression analysis was applied to test the impact of GxE on VTE risk after interaction terms were created between genetic and VTE risk factors [diabetes mellitus (DM), cancer, chronic kidney diseases (CKD), coronary artery diseases (CAD), migraine, depression, obesity, total cholesterol (TC), low-density lipoprotein cholesterol (LDL-C), high density lipoprotein (HDL-C), triglyceride (TG), and smoking].

**Results:** Interestingly, the rs121909567 (SERPINC1, ATBp3 mutation) SNP was not present in the general population at all. However, the risk allele frequency was 1% among the Roma population, which might suggest a founder effect in this minority. This polymorphism multiplicatively interacted with CAD, CKD, cancer, DM, depression, migraine, and obesity. Even though interactions were not statistically significant, the trend of interaction showed the probability of an incremental VTE risk among the Roma population. The risk of VTE was 4.7 times higher (*p* > 0.05) for Roma subjects who had ≥3 wGRS (median value) compared with individuals having lower wGRS values but lower for the general subjects (OR = 3.1 × 10^−8^). Additionally, the risk of VTE was 6.6 times higher in the Roma population that had ≥3 risk alleles (median value) than in individuals with the 0–1 risk allele, and the overall risk was much higher for the Roma population (OR = 6.6; *p* > 0.05) than for the general Hungarian population (OR = 1.5; *p* > 0.05). Five positive and significant GxE interactions were identified in the Roma population. The risk of VTE was higher among depressive Roma subjects who carried the risk variant rs2036914 (β = 0.819, *p* = 0.02); however, this interaction was not significant for the general subjects. The joint presence of high levels of LDL-C and rs2066865 (FGG) increased the VTE risk only among Roma individuals (β = 0.389, *p* = 0.002). The possibility of VTE risk increment, as a result of a multiplicative interaction between rs8176719 (ABO) and cancer, was identified, which was higher for the Roma population (β = 0.370, *p* < 0.001) than for the general population (β = −0.042, *p* = 0.6). The VTE risk increased in the Roma population (β = 0.280, *p* = 0.001), but was higher in the general population (β = 0.423, *p* = 0.001) as a result of the multiplicative interaction between CAD and rs2036914 (F11). The presence of a multiplicative interaction between rs2066865 (FGG) and CAD increased the VTE risk for the Roma population (β = 0.143, *p* = 0.046) but not for the general population (β = −0.329, *p* < 0.001).

**Conclusions:** rs121909567 (SERPINC1, ATBp3) was confirmed as a founder mutation in the Roma population. Our study revealed some evidence on the burden of the joint presence of genetic and environmental risk factors on VTE, although the finding is highly subjected to the selection and observational biases due to the very small number of VTE cases and the observational nature of the study design, respectively. As a result of higher genetic load and GxE interactions, this minority Roma population is at higher risk of VTE than the general Hungarian population. Thus, our results suggest the need for an intensive search for the rs121909567 (SERPINC1; ATBp3) founder mutation, which might be an important factor for the assessment of thrombotic disease susceptibility among the Roma population. In addition, we strongly recommend further studies among a large number of VTE cases to explore the more precise impact of genetic and environmental risk factors on VTE in the study populations.

## Introduction

Venous thromboembolism (VTE) is a multifactorial disease that occurs due to a combination of environmental, behavioral, and genetic risk factors. It contributes to relatively high morbidity and mortality within a short period after its occurrence (1, 2). The genetic basis of VTE is robust, and 50–60% of VTE is attributed to genetics. Family and twin studies confirmed the contribution of inheritable factors to VTE risk (3–5). The risk of recurrent VTE hospitalization among individuals with affected families was 1.92-fold that of the general population (6). An estimated incidence rate of VTE in subjects of European ancestry was 1–2 per 1,000 person/year, of which ~60% of the cases presented with deep venous thrombosis (DVT), whereas the remaining 40% presented with pulmonary embolism (PE) with or without DVT (7).

The presence of chronic diseases such as cancer, diabetes mellitus (DM), chronic kidney disease (CKD), and coronary artery disease (CAD) increases the likelihood of VTE among individuals (8–12). Furthermore, other personal and environmental risk factors, such as migraine (13–15), obesity, cigarette smoking, depression, high levels of lipoprotein, and antithrombin deficiency, also increase VTE risk. Previously conducted studies indicated that the risk of VTE was two times higher for obese individuals than for normal weight individuals (BMI 20–24.9 kg/m^2^) (16–18). Additionally, the risk of PE increases 6-fold among obese individuals compared with normal weight individuals (19). VTE risk is 1.3–1.7 times higher among current cigarette smokers than among non-smokers (20–22). Similarly, cigarette smoking was related to an absolute risk increase of 24.3 VTE cases per 100,000 person/year (22).

Studies have indicated that depression is a plausible risk factor for VTE (23–25); in a cohort study, Lee et al. revealed that the risk of VTE was 1.38 times higher among depressive than non-depressive individuals (23). Another systematic review and meta-analysis study showed that the risk of VTE was 1.3 times higher among depressed subjects than among non-depressed subjects (24).

In addition, a high level of low-density lipoprotein cholesterol (LDL-C) contributes to the occurrence of VTE. González-Ordóñez et al. reported that the risk of VTE was 2-fold higher among individuals with a high level of LDL-C than among individuals with a normal level of LDL-C (26). Another meta-analysis study of randomized control trials (RCTs) indicated that the risk of VTE was reduced among patients who received statin treatment for a high level of LDL-C (27). However, a cohort study reported no statistically significant association between lipoproteins [triglyceride (TG) and LDL-C] and VTE risk (28).

Furthermore, studies have found that antithrombin deficiency plays an important role in the pathogenesis of VTE. Antithrombin is an important inhibitor of blood coagulation proteases; individuals with hereditary AT deficiency have elevated thrombotic risk (29–31). Studies have revealed that the mutation profile of the AT gene (SERPINC1) is heterogeneous (32–35). Formerly, it was found that the prevalence of ATBp3 mutation was relatively high in the Roma population, but not in the general Hungarian population (36).

Likewise, there are populations that are susceptible to cardiovascular diseases due to the coexistence of genetic and environmental risk factors. The Roma are the most marginalized ethnic group in Central–Eastern European countries, with an estimated population of 8–12 million. The Roma experience social exclusion, which intensely affects their health outcomes (37). A higher burden of disease, low life expectancy, low socioeconomic status, low education, and harmful behavior are common among Roma minorities (38–42). As a result, cardiovascular risk factors are prevalent in the Roma population (43, 44). On the other hand, due to the restriction of health-related data collection by ethnicity in the Hungarian Roma population (45), to date, there is no incidence or prevalence data of VTE for this population. However, recent studies indicate that the Roma population is at higher risk of VTE due to an elevated prevalence of metabolic syndrome (46) and several inheritable risk factors (47).

Our previous study concluded that the Roma population seems to have increased genetic susceptibility to VTE. Further investigation has also suggested the necessity of comparing the gene–environmental interaction (GxE) for VTE risk in the general Hungarian and Roma populations (47). Understanding how genetic and environmental risk factors interact provides insight for the early identification of risk groups within populations, allowing appropriate preventive and therapeutic measures to be taken (48, 49). To date, no GxE comparison study has been conducted in the general Hungarian and Roma populations. Thus, the main aim of our current study was to explore the interaction of environmental risk factors with six prothrombotic SNPs [(rs121909567 (SERPINC1), rs1799963 (F2), rs2036914 (F11), rs2066865 (FGG), rs6025 (F5), and rs8176719 (ABO)] (36, 47, 50). In addition, we aimed to investigate the distribution of the rs121909567 (ATBp3) mutation in the SERPINC1 gene in the Hungarian population, which accounts for the vast majority of AT deficiencies in the Hungarian population due to its founder effect.

## Methods and Materials

### Study Design

A total of 832 (415 Roma and 417 Hungarian generals) subjects were culled from a comprehensive database created from the data obtained from a complex health survey for comparative and association studies (51). A total of 801 (395 Roma and 406 general Hungarian) subjects who had complete genotype and phenotype data were selected from the current study to assess and compare GxE and VTE risk.

### Study Populations and Data Used

Recently, a complex, three-pillar (questionnaire-based, physical examination, laboratory investigations) health survey was carried out to develop a database for the comparative and association studies to explore the underlying reasons for the very unfavorable health of Roma individuals when compared with the general population, especially as regards their high burden of cardiometabolic diseases. Details of the survey and the database created were published recently (51). Briefly, Roma subjects were recruited randomly from two counties (Szabolcs- Szatmár-Bereg and Hajdú-Bihar) in northeastern Hungary, the place where the Hungarian Roma are predominantly found and where the majority of the segregated Roma colonies are located. The reference group (representing the general population) included randomly selected individuals who lived in private households in the same counties of northeastern Hungary. Individuals aged 20–64 years were included in both groups. Demographic and anthropometric characteristics of the study populations, as well as laboratory data, were published previously (51).

Considering previously published reports on environmental and personal lifestyle risk factors found to affect VTE risk (8, 9, 12, 24, 52–54), the following data were extracted from the database:

- For chronic non-communicable diseases (cancer, DM, CAD, and CKD) proven to be risk factors for VTE (8, 12, 55), data were collected by a self-report questionnaire that assessed the history of experiencing those chronic diseases in the last 12 months before the survey. Respondents who replied “Yes” to the question regarding those chronic diseases were considered diseased, and otherwise not. Those respondents who answered “Yes” for the current smoking status were considered smokers. Similarly, the survey questionnaires assessed VTE through three questions: (1) Did you have thrombosis in the last 12 months? (2) Have you been diagnosed with thrombosis? (3) Have you received hospital treatment for thrombosis? Consequently, if the respondents replied “Yes” to either of those questions, we considered them “VTE cases,” whereas those who replied “No” were considered “non-cases.”
- Lipid (total cholesterol, LDL-C, high density lipoprotein, and TG) levels were measured.
- Other behavioral (smoking status) and psychosocial status data (depression and migraine histories), and
- Anthropometric measures (BMI and WC) were obtained.

### DNA Isolation

A MagNA Pure LC system (Roche Diagnostics, Basel, Switzerland) with a MagNA Pure LC DNA Isolation Kit-Large Volume was used to isolate DNA from the blood sample according to the instructions of the manufacturer. Extracted DNA was eluted in a 200 μl MagNA Pure LC DNA isolation kit-large volume elution buffer.

### SNPs Selection and Genotyping

Six SNPs, five prothrombotic SNPs [rs1799963 (F2), rs6025 (F5, Leiden), rs2066865 (FGG), rs2036914 (F11), and rs8176719 (ABO)] from our previous study (47), due to their confirmed and large effect size, and the rs121909567 SNP in the SERPINC1 gene, from the so-called antithrombin Budapest3 (ATBp3 mutation) study (36), were included in the present study. ATBp3 is the common cause of antithrombin (AT) deficiency in Hungary, and the founder effect of this mutation was previously considered in the Roma population (36, 56).

The assay design and genotyping were performed by Karolinska University Hospital, Stockholm, Sweden Mutation Analysis Core Facility (MAF). A MassARRAY platform (Sequenom, CA, USA) with iPLEX Gold chemistry was used for genotyping. Quality control, validation, and concordance analysis were conducted by MAF.

### Genetic Risk Score Computations

The weighted and the unweighted genetic risk scores (wGRS, uGRS) were computed to identify the combined effect of the included SNPs on VTE risk. In the GRS, the individuals were assigned a score based on the number of risk alleles they carried. Consequently, “0,” “1,” and “2” codes were given for the absence of risk alleles and heterozygosity and homozygosity for risk alleles, respectively. When the risk allele was found to be protective, the coding for the homozygous risk allele became “0,” whereas it became “2” for the other homozygous allele (47). Accordingly, uGRS was simply computed by adding all risk alleles in the loci assuming that all alleles had the same effect. However, wGRS was computed under the assumption that SNPs with larger effects contributed more to the GRS. Weights were derived from the risk coefficient because each allele depended on the odds ratio (OR) reported in the former genetic association study (50). For this study, only five SNPs that had an effect size in the previously conducted study were included for computing wGRS. Median values of wGRS and uGRS were used to compare the association between genetic risk score and VTE risk factors in the study populations.

### Statistical Analysis

Statistical tests were computed using IBM SPSS Version 25 statistical software. The Shapiro–Wilk normality test was used to test the distribution of quantitative variables. Non-normally distributed variables were transformed using a two-step Templeton's transforming approach (57). The presence of Hardy–Weinberg equilibrium (HWE) and allele frequency differences of all included SNPs in the two study populations were evaluated by using PLINK statistical software Version 1.9. A Bonferroni multiple testing was employed to prevent the problem associated with multiple comparisons. In our study, the level of significance for allele frequencies and individual SNPs comparison in the study populations was declared at an α level of 0.0083 (*n* = 6, α = 0.05; 0.05/6 = 0.0083).

Logistic regression analysis was used to determine the associations between individual SNPs, environmental risk factors, and VTE. A multivariate linear regression analysis with 95% CI was used to test the impact of GxE on VTE risk. An interaction term was created between each SNP and environmental risk factors to assess their combined effect on VTE risk.

Three categories of ORs were defined based on the GxE assumption models: subjects who were unexposed to environmental risk factors and free from risk variants (also known as wild type) (E=G=0), were used as reference groups (OR_00_); OR_11_ represented subjects with both genetic and environmental risk factor exposure (E=G=1); OR_10_ = subjects exposed to environmental risk factors but not with genetic risk (E=1, G=0); and OR_01_ = subjects with only genetic exposure but not environmental risk exposure (E=0, G=1). Thus, under the multiplicative interaction model, if OR_11_ = OR_01_ × OR_10_, there was no interaction between genetic and environmental risk factors; however, if OR_11_ ≠ OR_01_ × OR_10_, there was a multiplicative interaction between the given environmental risk factors and genetic risk factors, which was either a synergistic interaction (OR_11_ > OR_01_ × OR_10_) or an antagonistic interaction if OR_11_ < OR_01_ × OR_10_ (58, 59). For the additive model, no interaction was concluded based on the null hypothesis (H_0_) of OR_11_ = OR_01_ + OR_10_ – 1; however, the interaction would be considered synergistic when OR_11_ > OR_01_ + OR_10_ – 1 or antagonistic if OR_11_ < OR_01_ + OR_10_ – 1 (58, 59).

During the analysis, the general Hungarian and Roma population samples were combined, and ethnicity was included as a variable in the model to differentiate its effect on VTE risk. For this study, the general Hungarian population was used as a reference population to compare the GxE interaction and VTE risk in the two populations. All analyses were adjusted for age, and the reported *p* values were two-sided. An α level of 0.05 was used to define the statistical significance.

### Ethical Approval

The Committee of the Hungarian Scientific Council on Health Research approved the protocol (61327-2017/EKU). Written informed consent was obtained from all study subjects.

## Results

### Characteristics of the Study Participants

The 801 study subjects (395 Roma population and 406 Hungarian generals) who had complete genotype and phenotype data were involved in this study. The female proportion in the Roma sample was higher than that in the Hungarian general sample (55.4 vs. 73.9%; *p* < 0.001). Further details on the study population characteristics are presented in Table 1.

**Table 1:** Characteristics of the study participants selected from the database of the complex survey.

**Characteristics**	**General Hungarian** **(*N* = 406)**	**Roma** **(*N* = 395)**	***p***
	**(Mean, 95% CI)**	**(Mean, 95% CI)**	
Age (years)	44.3 (43.1–45.5)	43.5 (42.2–44.7)	0.35
**Female (%)**	**55.4**	**73.9**	**<0.001**
BMI (kg/m^2^)	27.2 (26.7–27.7)	27.5 (26.8–28.2)	0.48
WC (cm)	96.0 (94.5–97.5)	95.0 (93.3–96.7)	0.38
Cholesterol (mmol/l)	5.0 (4.9–5.1)	4.9 (4.8–5.0)	0.45
LDL-C (mmol/l)	3.1 (3.0–3.2)	3.1 (3.0–3.2)	0.31
**HDL-C (mmol/l)**	**1.4 (1.3–1.4)**	**1.3 (1.2–1.3)**	**<0.001**
TG (mmol/l)	1.6 (1.5–1.7)	1.7 (1.6–1.9)	0.29
FBG (mmol/l)	5.3 (5.1–5.5)	5.2 (5.0–5.4)	0.51

*BMI, Body mass index; WC, waist circumference; LDL-C, Low-density lipoprotein cholesterol; HDL-C, High density lipoprotein cholesterol; TG, Triglycerides; FBS, Fasting blood glucose. Significance differences are highlighted in bold*.

### VTE Morbidity in the Study Populations

In the current study, only 6 (1.5%) and 12 (3.0%) of the Hungarian general and Roma populations, respectively, reported VTE during the survey. The proportion of VTE cases was higher among the Roma population; in particular, the VTE risk was higher among female subjects (Table 2).

**Table 2:** Distribution of VTE cases by population and sex among the study subjects.

**Sex**	**General Hungarian (*****N*** **=** **406)**		**Roma (*****N*** **=** **395)**	
	**Yes (%)**	**No (%)**	**Yes (%)**	**No (%)**
Male	4 (2.2)	177 (97.8)	4 (3.9)	99 (96.1)
Female	2 (0.9)	223 (99.1)	8 (2.7)	284 (97.3)
Total	6 (1.5)	400 (98.5)	12 (3.0)	383 (97.0)

### Allele Frequency Comparison of the Study Populations

All included SNPs were checked for HWE; no significant deviation from HWE was observed in the study populations. The allele frequencies of the five prothrombotic SNPs [(rs1799963 (F2), rs2036914 (F11), rs2066865 (FGG), rs6025 (F5), and rs8176719 (ABO)] of the current study (Table 3) were not significantly different from those of our previous study (47). Before multiple corrections testing, allele frequencies of rs121909567 (SERPINC1) and rs1799963 (F2) were significantly different among the two populations. However, after adjustment for multiple testing using Bonferroni correction rs121909567 (SERPINC1) allele frequency remains significant among the study populations. The genotype distribution was used to calculate the allele frequencies in the study populations.

**Table 3:** Comparison of effect allele frequencies (%) in the general Hungarian and Roma populations.

**Gene**	**SNPs**	**Risk** **allele**	**General** **Hungarian** **(*N* = 406),** **frequency (%)**	**Roma** **(*N* = 395),** **frequency (%)**	***p***
**SERPINC1**	**rs121909567**	**A**	**0 (0%)**	**0.01 (1%)**	**<0.001**
F2	rs1799963	A	0.02 (2%)	0.01 (1%)	0.04
F11	rs2036914	C	0.54 (54%)	0.51 (51%)	0.2
FGG	rs2066865	A	0.23 (23%)	0.28 (28%)	0.05
F5	rs6025	T	0.07 (7%)	0.09 (9%)	0.2
ABO	rs8176719	G	0.47 (47%)	0.48 (48%)	0.6

*Significant differences are highlighted in bold*.

### Comparison of GRS

**Figure 1:**
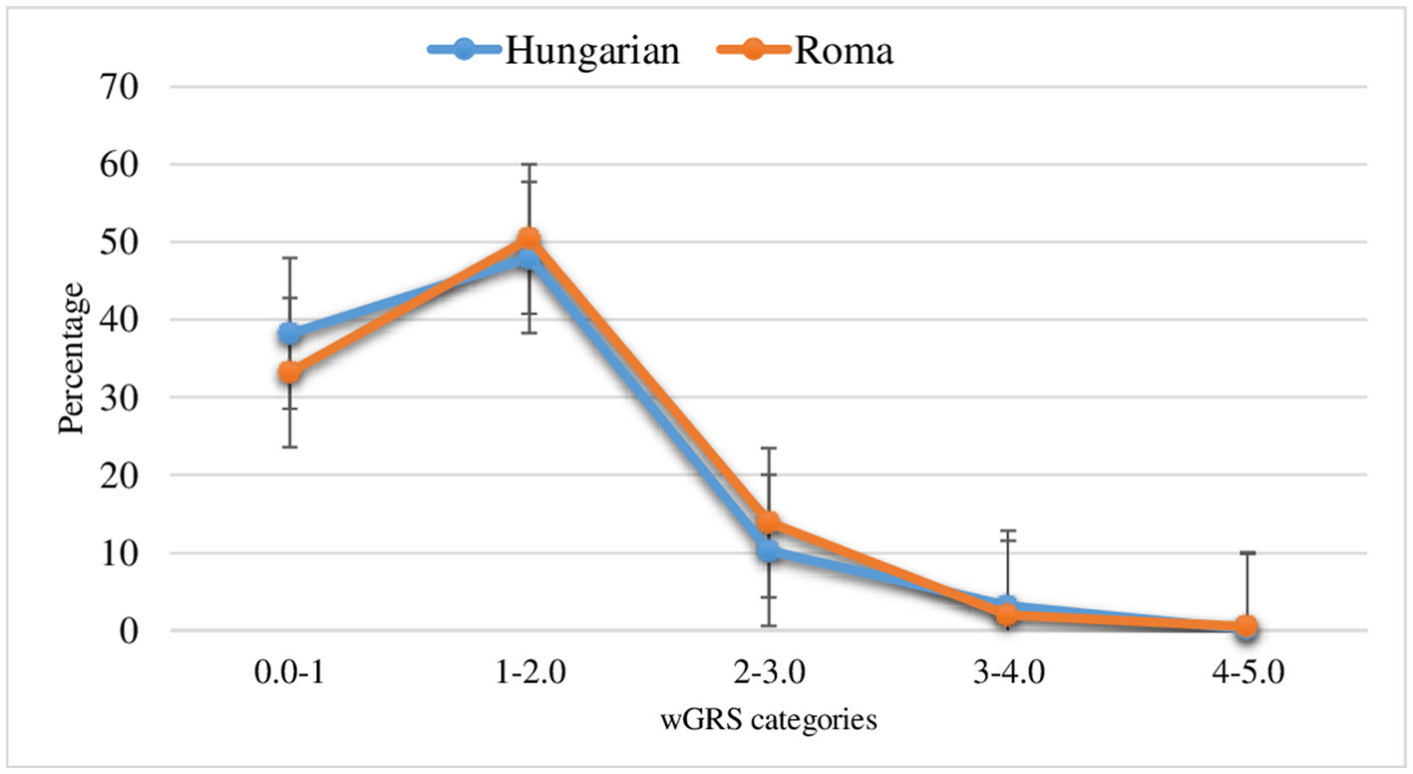
Distribution of wGRS in the general Hungarian and Roma population (The error bars indicate the standard error of the mean).

**Figure 2:**
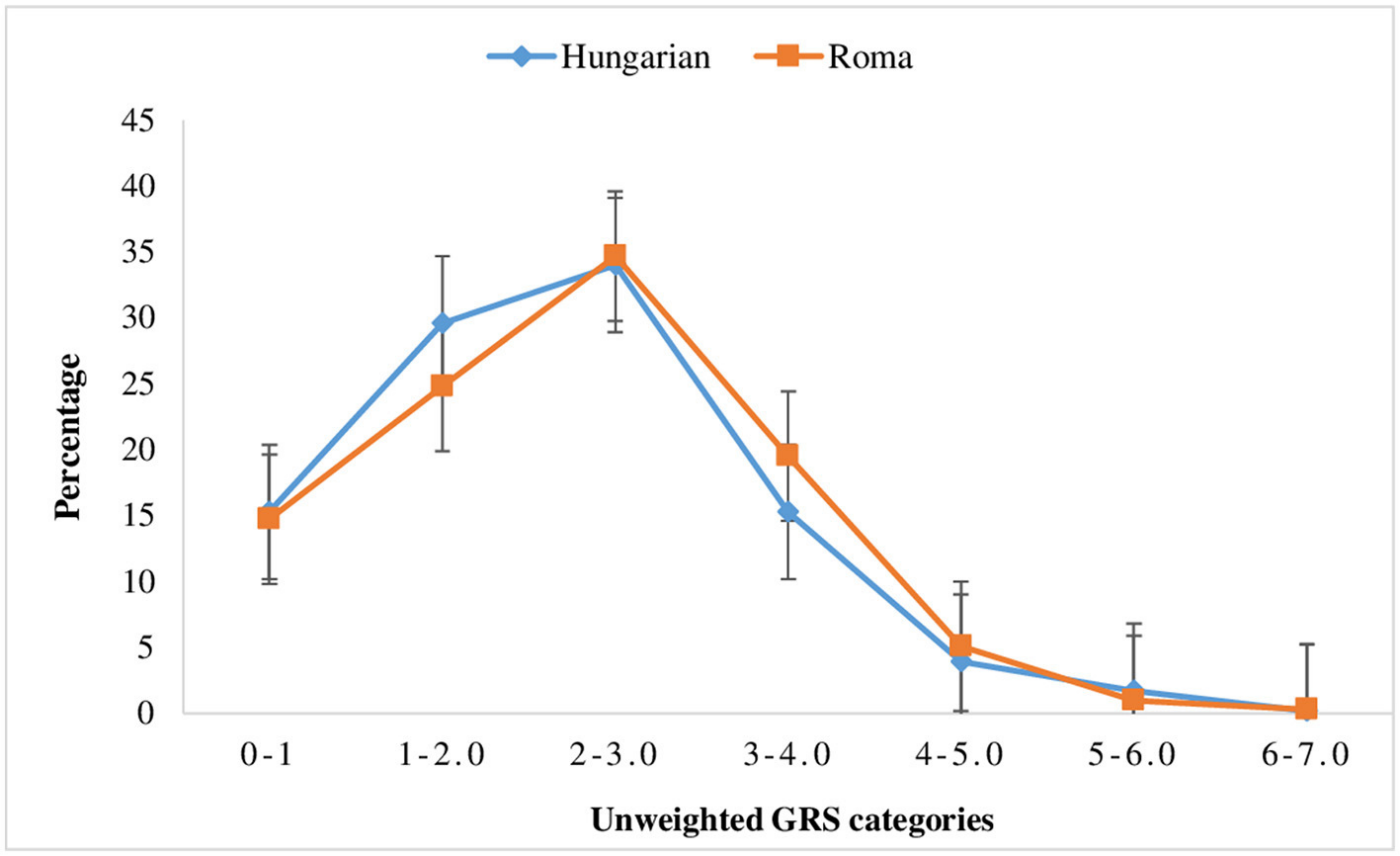
Distribution of un-weighted genetics risk scores in the general Hungarian and Roma population (the error bars indicate the standard error of the mean).

In this study, the wGRS was computed using only five SNPs (Supplementary Table 1), which showed a strong association with VTE from previously conducted studies (47, 50). Due to the absence of a published external weight (60) for rs121909567 (ATBp3 mutation), the wGRS computation did not consider this particular SNP. The wGRS ranged from 0.0 to 4.7 and 0.0 to 4.6 for the general Hungarian and Roma populations, respectively. The mean wGRS was 1.8 ± 0.8 (95% CI [1.7, 1.9]) for the general Hungarian population and 1.9 ± 0.76 (95% CI [1.8, 1.9]) for the Roma population (Figure 1). The uGRS was calculated for six SNPs, and it ranged from 0.0 to 7.0 for both populations, with a mean of 2.7 ± 1.2; 95% CI [2.6, 2.8] for the general Hungarian population and 2.8 ± 1.2; 95% CI [2.7, 2.9] for the Roma population (Figure 2).

Although it was not statistically significant, the risk of VTE based on the joint effect of prothrombotic risk alleles was higher for Roma subjects who had ≥3 wGRS (OR = 4.74; 95% CI [0.45, 50.3]) than for individuals with 0–1 wGRS values, but not for the general Hungarian population (OR = 3.1 × 10^−8^ *p* > 0.05). Additionally, the risk of VTE was 6.6 times higher in the Roma population who had ≥3 risk alleles than in individuals with 0–1 risk alleles, and the risk was much higher for the Roma population (OR = 6.6; *p* > 0.05) than for the general Hungarian population (OR = 1.5; *p* > 0.05).

### Associations of Individual SNPs With VTE

As shown in Table 4, only the Leiden mutation (rs6025) and rs2066865 (FGG) were significantly associated with VTE risk and found to be nominally significant for the Roma population, but not for the general population. Study subjects who had homozygous risk alleles for the FGG gene were 5.9 times more at risk for VTE than subjects without a risk allele (OR = 5.9; 95% CI: [1.23, 28.4]). Furthermore, people with the heterozygous risk allele of the Leiden mutation (rs6025) were 3.8 times more likely to develop VTE than individuals without the risk allele, and the risk was higher for the Roma population. However, this significance does not exist (*p* > 0.0083) after we adjust the test using Bonferroni multiple correction testing. Furthermore, our study reveals that some individuals who had VTE were carriers for homozygous risk variants of multiple SNPs. In our study, three Roma subjects who had VTE were carriers of the homozygous risk variant rs2036914 (F11), and another three individuals carried the homozygous variant of rs2066865 (FGG), as depicted in Table 4. However, two out of the six Roma subjects, who had VTE and were carriers for homozygous risk variants of rs2036914 (F11) and rs2066865 (FGG), also carried homozygous risk variants of both SNPs.

**Table 4:** Comparisons of associations between individuals SNPs and VTE in the general Hungarian and Roma populations.

		**VTE cases**					
		**General Hungarian**			**Roma**		
**Gene (SNPs)**	**Genotype**	**Yes**	**No**	**OR (95% CI)**	**Yes**	**No**	**OR (95% CI)**
SERPINC1 (rs121909567)	G G	6 (1.5)	400 (98.5)	NA^‡^	12 (3.1)	372 (96.9)	1.00
	G A	0 (0.0)	0 (0.0)		0 (0.0)	11 (100)	2.2E-8
F2 (rs1799963)	G G	6 (1.5)	384 (98.5)	1.00	12 (3.1)	377 (96.9)	1.00
	G A	0 (0.0)	16 (100)	4.4E-8	0 (0.0)	6 (100.0)	2.9E-8
F11 (rs2036914)	T T	1 (1.2)	83 (98.8)	1.00	3 (3.0)	97 (97.0)	1.00
	C T	4 (2.0)	201 (98.0)	1.4 (0.13, 13.9)	6 (3.2)	184 (96.8)	0.96 (0.22, 4.2)
	C C	1 (0.9)	116 (99.1)	0.49 (0.03, 9.5)	3 (2.9)	102 (97.1)	1.15 (0.21, 6.4)
FGG (rs2066865)	G G	2 (0.9)	233 (99.1)	1.00	5 (2.4)	201 (97.6)	1.00
	G A	3 (2.0)	149 (98.0)	2.5 (0.39, 16.2)	4 (2.5)	156 (97.5)	0.95 (0.24, 3.8)
	A A	1 (5.3)	18 (94.7)	5.6 (0.43, 73.9)	3 (10.3)	26 (89.7)	5.9 (1.23, 28.4)^*^
F5 (rs6025)	C C	4 (1.1)	347 (98.9)	1.00	8 (2.4)	323 (97.6)	1.00
	C T	2 (3.7)	52 (96.3)	3.5 (0.57, 21.3)	4 (6.7)	56 (93.3)	3.8 (1.01, 14.2)^*^
	T T	0 (0.0)	1 (100)	1.1E-7	0 (0.0)	4 (100.0)	1.1E-8
ABO (rs8176719)	C C	5 (4.2)	114 (95.8)	1.00	5 (4.7)	101 (95.3)	1.00
	C DEL	1 (0.5)	193 (99.5)	0.11 (0.01, 0.9)	5 (2.5)	192 (97.5)	0.49 (0.13, 1.9)
	DEL DEL	0 (0.0)	93 (100.0)	1.6E-8	2 (2.2)	90 (97.8)	0.43 (0.08, 2.5)

‡ *No risk allele found in the general Hungarian for rs121909567 SNP*,

* *p < 0.05*.

### GxE and VTE Risk

As indicated in Supplementary Table 2, multivariate linear regression analysis was performed to explore the impact of GxE on VTE risk among the study subjects. The finding of the GxE and VTE risk in our study was reported using the standardized beta value of the multivariate linear regression analysis.

We found several statistically significant multiplicative interactions on VTE risk for the majority of the included SNPs, such as rs2036914 (F11), rs2066865 (FGG), rs6025 (F5, Leiden), and rs8176719 (ABO). Since the ATBp3 mutation was not present in the general population, regression analysis was only performed for the Roma population. Although the trend of their relationships indicated the possibility of higher VTE risk, the multiplicative interaction was not statistically significant for the SERPINC1 (rs121909567) and F2 (rs1799963) genes (*p*-value for interaction >0.05). According to the regression coefficient of the multivariate linear regression analysis, the likelihood of VTE was higher among the study subjects who had cancer, DM, CAD, and CKD, plus experienced migraine and depression in addition to their genetic susceptibility to VTE. Furthermore, cigarette smoking, a high level of LDL-C, and obesity increased the VTE risk for the study subjects.

**Figure 3:**
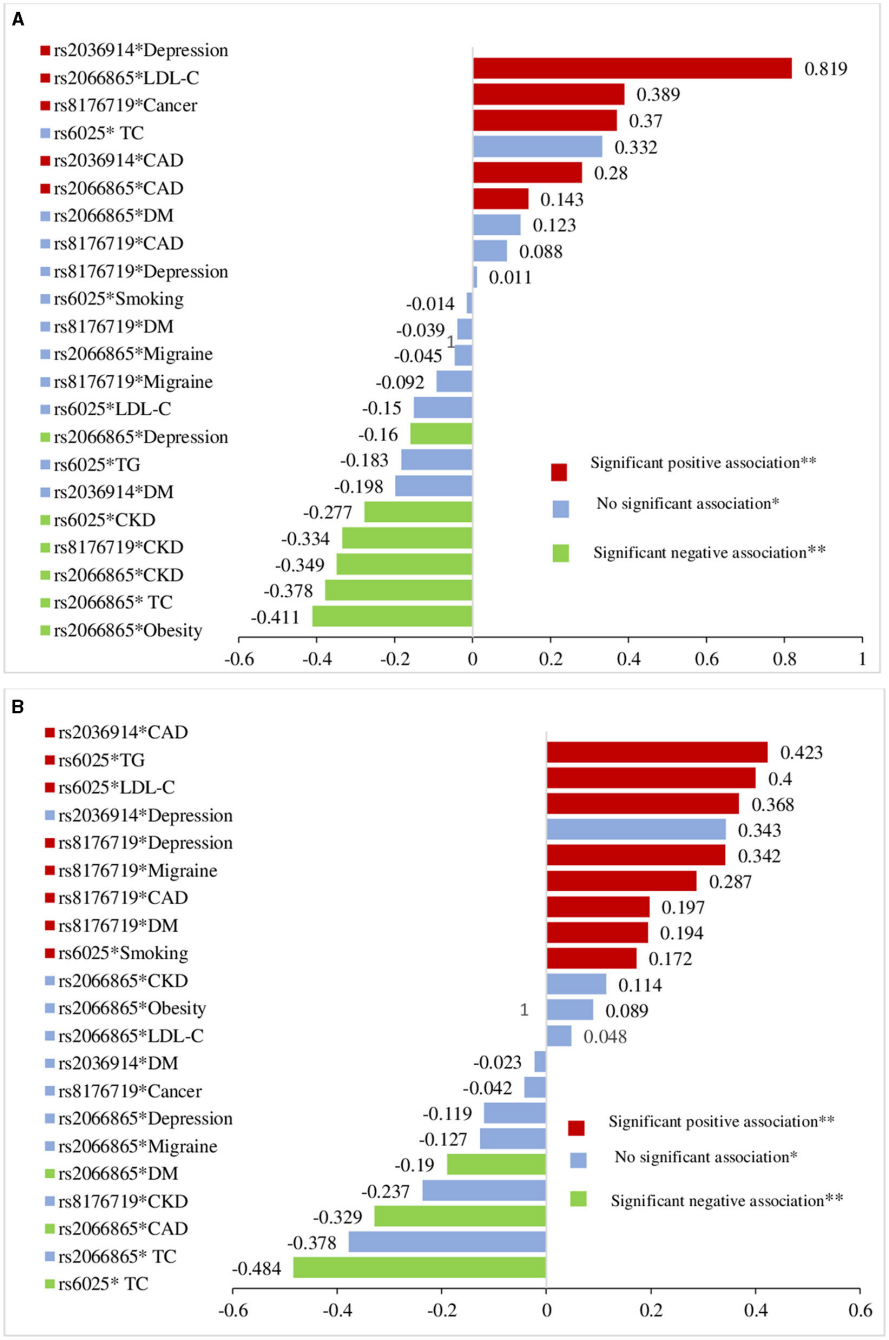
Comparison of G × E on the VTE risk among Roma population **(A)** and general Hungarian **(B)** based on standardized linear regression coefficients from multivariate linear regression analysis after interaction terms included between genes and VTE risk factors.

Figure 3 shows that the standardized beta (β) values of the GxE and VTE risk from the linear regression analyses were statistically significant in both the study populations. The observed multiplicative interaction and VTE risk were bidirectional: a positive beta (β) value indicated a VTE risk increment as a result of GxE (red color), whereas a negative beta (β) value indicated the reverse (green color).

The risk of VTE was higher (β = 0.819, *p* = 0.02) among depressive Roma individuals with the rs2036914 risk variant, and it was statistically significant, but not for the general Hungarian individuals (β = 0.343, *p* = 0.33). The presence of high levels of LDL-C and the rs2066865 (FGG) risk variant makes Roma subjects at higher risk of VTE (β = 0.389, *p* = 0.002); however, the joint presence of those risk factors did not increase the VTE risk in the general subjects (β = 0.048, *p* = 0.70). The existence of a multiplicative interaction between CAD and rs2036914 (F11) increases the VTE risk among both the populations: the Roma population (β = 0.280, *p* = 0.001) and the general Hungarian population (β = 0.423, *p* = 0.001).

As a result of the multiplicative interaction between rs2066865 (FGG) and CAD, VTE risk was higher for the Roma population (β = 0.143, *p* = 0.046), but not for the general Hungarian population (β = −0.329, *p* < 0.001). Nonetheless, the interaction between this particular SNP and depression was not positively related to VTE risk (β = −0.160, *p* = 0.046) for the Roma or general Hungarian population (β = −0.119, *p* = 0.11). The interaction between rs6025 (F5, Leiden) and smoking (β = 0.172, *p* = 0.008), and also Leiden and LDL-C (β = 0.368, *p* = 0.001) increased the risk of VTE for the general population only, but not for the Roma population (β = −0.014, *p* = 0.86 and β = −0.150, *p* = 0.55, respectively).

Our study also identifies the higher risk of VTE as a result of a multiplicative interaction between rs8176719 (ABO) and cancer, and the risk was higher for the Roma population (β = 0.370, *p* < 0.001) than for the general Hungarian population (β = −0.042, *p* = 0.6). Nevertheless, the interaction of rs8176719 (ABO) with CAD, (β = 0.197, *p* = 0.009), migraine (β = 0.287, *p* = 0.001), and depression (β = 0.342, *p* < 0.001) significantly increased VTE risk only for the general Hungarian population. The risk of VTE was higher for general Hungarian subjects (β = 0.194, *p* < 0.01) who had diabetes mellitus and non O blood type, but not for the Roma subjects (β = −0.039, *p* = 0.63) (Figure 3).

### Association Between GRS and VTE Risk Factors

Weighted GRS was computed for five SNPs that were strongly associated with VTE in former studies. Logistic regression analysis was performed to test the relationships between wGRS, uGRS, and VTE risk factors. The effects of the combined genetics risk factors and a high level of LDL-C, migraine, and current cigarette smoking were additive and statistically significant for the Roma population. As revealed in Table 5 below, a high level of LDL-C (OR = 3.2; 95% CI [1.2, 8.8]) and migraine (OR = 3.9; 95% CI [1.1, 12.3]) increased the risk of VTE in the Roma population but not in the general population.

**Table 5:** Association between wGRS and VTE risk factors.

	**General Hungarian** **(*****N*** **=** **406)**		**Roma (*****N*** **=** **395)**	
**VT risk factors**	**OR (95% CI)**	***p***	**OR (95% CI)**	***p***
TC^a^	1.6 (0.3–8.0)	0.57	1.5 (0.5–4.8)	0.47
**LDL-C** ^**b**^	1.8 (0.4–9.1)	0.47	**3.2 (1.2–8.8)**	**0.02**
HDL-C^c^	0.7 (0.1–3.8)	0.69	1.2 (0.4–3.7)	0.81
TG^d^	3.1 (0.5–18.9)	0.22	2.3 (0.6–8.6)	0.22
**Migraine** ^**e**^	3.0 (0.7–13.8)	0.15	**3.9 (1.1–12.3)**	**0.03**
Currently smoking cigarettes^f^	1.0 (0.5–2.0)	0.91	1.9 (0.9–3.8)	0.06
**Cessation of smoking for** ** <1 year**^**g**^	1.1 (0.1–8.9)	0.95	**0.1 (0.0–0.9)**	**0.04**
**Cessation of smoking for** **>1 year**^**h**^	1.9 (0.4–8.2)	0.42	**0.2 (0.1–0.6)**	**0.004**
Obesity (BMI > 30 kg/m^2^)	1.2 (0.5–3.0)	0.64	1.7 (0.7–3.8)	0.2

a *Total cholesterol ≥ 5.2 mmol/L*.

b *LDL-C level ≥ 3.4 mmol/L*.

c *HDL-C <1.3 mmol/L*.

d *TG ≥1.7 mmol/L*.

e *Reported as they had migraine in the past 12 month*.

f *Currently smoking cigarette*.

g *Ever smoked cigarette but stopped smoking for less than a year*.

h *Ever smoked cigarette but stopped smoking for more than a year*.

*Significant differences are highlighted in bold*.

Furthermore, this study indicated that the risk of VTE was reduced among study participants who had ever smoked cigarettes but quit cigarette smoking <1 year (OR = 0.1; 95% CI [0.0, 0.9]) and more than 1 year (OR = 0.2; 95% CI [0.1, 0.6]). The risk of VTE among Roma individuals who had >3.0 wGRS, but not currently smoking was reduced by 10 and 20%, respectively, but not for the general Hungarian individuals. However, cigarette smoking did not have a statistically significant additive effect on the VTE risk (OR = 1.9; 95% CI [0.9, 3.8]).

For the unweighted GRS, only a high level of LDL-C (OR = 2.2, 95% CI [1.0, 4.7]) was associated with VTE risk in an additive model, and the risk was higher for the Roma population but not for the general Hungarian population (OR = 1.5; 95% CI [0.7, 3.2]) (Table 6).

**Table 6:** Association between uGRS and VTE risk factors.

**VT risk factors**	**General Hungarian** **(*****N*** **=** **406)**		**Roma (*****N*** **=** **395)**	
	**OR (95%CI)**	***p***	**OR (95% CI)**	***p***
TC^a^	1.5 (0.3–7.7)	0.60	1.5(0.5–4.8)	0.48
**LDL-C** ^**b**^	1.5 (0.7–3.2)	0.36	**2.2 (1.0–4.7)**	**0.04**
HDL-C^c^	0.7 (0.1–3.6)	0.62	1.2 (0.4–3.8)	0.79
TG^d^	3.3 (0.5–20.0)	0.19	2.3 (0.6–8.5)	0.22
Migraine^e^	1.0 (0.5–2.3)	0.96	1.4 (0.7–2.7)	0.32
Currently smoking cigarettes^f^	1.1 (0.6–1.8)	0.76	1.5 (0.9–2.4)	0.09
Cessation of smoking for <1 year^g^	1.6 (0.3–8.9)	0.6	0.2 (0.02–1.8)	0.2
Cessation of smoking for >1 year^h^	1.2 (0.5–2.8)	0.7	1.1 (0.4–3.1)	0.9
Obesity (BMI >30 kg/m^2^)	1.6 (0.9–3.1)	0.1	1.1 (0.6–1.8)	0.8

a *Total cholesterol ≥ 5.2 mmol/L*.

b *LDL-C level ≥ 3.4 mmol/L*.

c *HDL-C <1.3 mmol/L*.

f *TG≥1.7 mmol/L*.

e *Reported as they had migraine in the past 12 month*.

f *Currently smoking cigarette*.

g *Ever smoked cigarette but stopped smoking for less than a year*.

h *Ever smoked cigarette but stopped smoking for more than a year*.

*Significant differences are highlighted in bold*.

## Discussion

A total of 395 Roma and 406 general subjects with full clinical and genotype records were involved in the analyses. We assessed interactions between six prothrombotic SNPs [rs121909567 (SERPINC1), rs1799963 (F2, prothrombin G20210A), rs6025 (F5, Leiden), rs2066865 (FGG), rs2036914 (F11), and rs8176719 (ABO)] and environmental factors that were proven to be risk factors for VTE (8–10, 12, 16–24, 53, 61). Our study is the first to investigate and compare GxE in populations of Hungarians (Roma vs. general) and found evidence for higher GxE and VTE risk among Roma individuals.

The current study revealed that GxE and VTE risk was predominantly common among a group of populations with a specific SNP but not among others; a statistically significant multiplicative interaction was observed between the rs8176719 (ABO) gene and diabetes mellitus, migraine, depression, and CAD for the general Hungarian population, but only with cancer for the Roma population. The coefficient of multiplicative interactions of diabetes mellitus and rs8176719 (ABO) was positive and significant, indicating that non-O blood type general Hungarian individuals who had diabetes mellitus were more likely to have a higher VTE risk. This finding was supported by a systematic review and meta-analysis of cohort studies that indicated that diabetes mellitus increases the VTE risk 1.4 times compared with subjects without diabetes mellitus (62). Conversely, another systematic review and meta-analysis of case-control studies (63) reported no significant association. This discrepancy might be due to the study design and sample size they considered for analysis.

Our study demonstrated that subjects experiencing migraine and non-O blood types were at higher risk of developing VTE. This finding is in line with studies that indicated that the presence of migraine increased VTE risk by 1.3-(14), 1.5-(13, 15), and 2.5-(61) fold. The present study also suggests that VTE risks increased among depressive individuals with risk variants for rs2036914 (F11) among Roma subjects and rs8176719 (ABO) among general Hungarian subjects. This result is in accordance with previous investigations indicating that depression increases VTE risk (23, 24). Moreover, the risk of developing VTE was six to seven times higher in cancer patients (7, 64, 65). This result was consistent with our study findings which reveal that the VTE risk is higher for the Roma subjects who had cancer.

The VTE risk was higher among non-O blood type Roma subjects who had cancer as well. Our study also confirmed other studies (10, 66) which showed that the presence of cancer increased the risk of VTE in addition to other VTE risk factors. Further studies on cancer and prothrombotic genotypes point out that VTE risk increased by 11–12-fold as a result of the simultaneous presence of cancer and rs8176719 (ABO) risk variant (67, 68). The authors also found that 39% (67) and 30% (68) of VTE risk was attributed to the joint presence of cancer and non-O-blood type.

An earlier study (64) found that the presence of cancer and the Leiden mutation (rs6025) variant increases the VTE risk by 2-fold; this concurred with our result, although the association was not statistically significant. The lack of significance might be due to the small proportion of individuals with cancer and VTE, the study design, and the relatively small sample size.

The multiplicative interaction coefficient of Leiden mutation (rs6025) and cigarette smoking was positive and statistically significant. The possibility of VTE risk was higher in the general Hungarian subjects who smoke cigarettes and carried the risk variant for the Leiden mutation (rs6025). Prior studies also revealed that the combined effect of rs6025 (F5) and smoking increased the risk of VTE (69, 70). A large population-based case-control study also supported our finding, where the joint effect of rs6025 (F5) and current smoking resulted in a 5-fold increased VTE risk (71). Another cohort study revealed that the simultaneous presence of smoking in addition to rs6025 (F5) increased the VTE risk by 51 and 10% at 10 years for homozygous and heterozygous risk variants, respectively (72). Crous-Bou et al. also found an additive interaction between prothrombotic SNPs and smoking that increased VTE risk (53).

Study subjects with coronary artery diseases and rs2036914 (F11) or rs8176719 (ABO) were at higher risk of VTE than subjects without CAD and those variants. The presence of an interaction between rs2036914 (F11) and CAD increased the VTE risk among both the study populations who carried those risk factors. Sejrup et al. found that myocardial infarction patients with ≥1 risk allele at rs2036914 (F11) had a 1.8-fold higher risk of PE (73). Furthermore, the risk of VTE was 1.5-fold higher among individuals with non-O blood type and myocardial infarction (73). Our finding was also consistent with this study.

Additionally, the current study also presented an additive interaction between a high level of LDL-C, migraine, current cigarette smoking, and ≥3 wGRS value. The risk of VTE increased by 3.2-fold for the Roma individuals with a high level of LDL-C and ≥3 risk alleles. This result was in line with a GWAS (74) which revealed that one standard deviation (SD) of elevated LDL-C was associated with an increased risk of VTE.

The finding of current cigarette smoking was in agreement with the study of Crous-Bouet al., in which the relationships between current smoking and VTE genetic factors were additive (53). In our study, individuals who had experience with migraine in addition to a wGRS value of ≥3 had 3.9 times higher risk for VTE than individuals with either a wGRS≥3 or migraine. This finding was in agreement with a study by Peng et al., who revealed that migraine headaches increased the risk of VTE (61).

Although the multiplicative interaction between rs121909567 (SERPINC1), rs1799963 (F2), and VTE risk factors was not statistically significant, and their regression coefficient indicated the possibility of higher VTE risk among individuals who have dual exposures. Interestingly, rs121909567 (SERPINC1) (ATBp3 mutation) multiplicatively interacted with CAD, CKD, cancer, DM, depression, migraine, and obesity. Even though their relationships were not statistically significant, the trend of interaction showed the probability of VTE risk increment among the Roma population. The lack of statistical significance between GxE and VTE risk for rs121909567 (SERPINC1) and rs1799963 (F2) might be due to the very small number of VTE cases.

Similarly, studies have shown that the prevalence of cardiovascular risk factors was higher in the Roma population (43, 44), and we found that in addition to environmental factors, genetic susceptibility contributes to high cardiovascular mortality/morbidity (75). In a relatively isolated population such as the Roma, the consanguinity rate is high (76); consequently, it was assumed that a founder mutation had an impact on the development of thrombotic diseases (36). A recent study also identified a high prevalence (2.74%) of ATBp3 mutation in the Roma population; however, no ATBp3 mutation was found in the general Hungarian population (56). This finding was in line with our study results, which revealed that ATBp3 mutations were found only in the Roma population but not in the general Hungarian population.

Altogether, rs121909567 (SERPINC1, ATBp3) was confirmed as a founder mutation among the Roma population. The present study also reveals some clues about the burden of the joint presence of genetic and environmental risk factors on VTE risk, although the finding was highly subject to selection and observational biases due to the very small number of VTE cases and the observational nature of the study design, respectively. As a result of higher genetic load and GxE interactions, this minority Roma population is at higher risk of VTE than the general Hungarian population. Thus, our results suggest that an intensive search for the rs121909567 (SERPINC1; ATBp3) founder mutation might be an important factor for the assessment of thrombotic disease susceptibility among the Roma population. In addition, we strongly recommend further studies among a large number of VTE cases to explore the more precise impact of genetic and environmental risk factors on VTE in the study populations.

## Data Availability Statement

The raw data supporting the conclusions of this article will be made available by the authors, without undue reservation.

## Ethics Statement

The studies involving human participants were reviewed and approved by the Committee of the Hungarian Scientific Council on Health Research (61327-2017/EKU). Written informed consent was obtained from all study subjects. The patients/participants provided their written informed consent to participate in this study.

## Author Contributions

RÁ: conceptualization. SN and SF: data handling, writing, and interpreting the results, and preparing the manuscript. SN and MM: statistical analysis. ZK, PP, and JS: data collection and management. SN, SF, ZB, and RÁ: review, editing, and finalizing the manuscript. All authors contributed to the article and approved the submitted version.

## Funding

This study was financed by the Stipendium Hungaricum Scholarship Programme of the Tempus Public Foundation, the European Union under the European Regional Development Fund (GINOP-2.3.2-15-2016-00005 and GINOP-2.3.2-15-2016-00039), the Hungarian Academy of Sciences (MTA11010 and TK2016-78) and the National Research, Development and Innovation Office, Hungarian Ministry of Innovation and Technology (Grant No. OTKA K116228). Project no. 135784 has also been implemented with the support provided by the National Research, Development and Innovation Fund of Hungary, financed under the K_20 funding scheme.

## Conflict of Interest

The authors declare that the research was conducted in the absence of any commercial or financial relationships that could be construed as a potential conflict of interest.

## Publisher's Note

All claims expressed in this article are solely those of the authors and do not necessarily represent those of their affiliated organizations, or those of the publisher, the editors and the reviewers. Any product that may be evaluated in this article, or claim that may be made by its manufacturer, is not guaranteed or endorsed by the publisher.
