# Supplementary material for: The Higher Prevalence of Venous Thromboembolism in the Hungarian Roma Population Could Be Due to Elevated Genetic Risk and Stronger Gene-Environmental Interactions
Source: Front Cardiovasc Med. 2021 Oct 26;8:647416. doi: 10.3389/fcvm.2021.647416 (PMC8576195; doi:10.3389/fcvm.2021.647416)
Supplement: Supplementary file 1 [file Data_Sheet_1.docx]

Supplementary Material

# Supplementary Tables

- 1. **Supplementary Table 1.** SNPs/related genes used in the weighted GRS computation, and weighting numbers with the original publication they were adopted from.

| **Gene** | **SNPs** | **Risk Allele** | **OR** | **Weighting Number (lnOR)** | **Reference** |
| --- | --- | --- | --- | --- | --- |
| F2 | rs1799963 | A | 2.78 | 1.02 | (42) |
| F11 | rs2036914 | C | 1.32 | 0.28 |  |
| FGG | rs2066865 | A | 1.56 | 0.44 |  |
| F5 | rs6025 | T | 3.79 | 1.33 |  |
| ABO | rs8176719 | G | 1.85 | 0.62 |  |

- 1. Supplementary table 2: Comparison of GxE on the VTE risk among Hungarian Roma and general populations based on standardized linear regression coefficients from multivariate linear regression analysis after interaction terms were included between SNPs and VTE risk factors

| **Gene** | **Roma** | | | | | | **general Hungarian** | | | | |
| --- | --- | --- | --- | --- | --- | --- | --- | --- | --- | --- | --- |
|  | **SNPs (GXE)** | **β** | ***p*-value** | **95%CI** | **ADr^2^** | **r^2^ change (*p*-value)** | **β** | ***p*-value** | **95%CI** | **ADr^2^** | **r^2^ change( *P*-value)** |
| SERPENC1‡ | rs121909567*DM | 0.009 | 0.94 | (-0.41-0.38) | 0.016 | 0.007(>0.05) |  |  |  |  |  |
|  | rs121909567*Cancer | 0.034 | 0.76 | (-0.43-0.59) |  |  |  |  |  |  |  |
|  | rs121909567*CKD | 0.06 | 0.65 | (-0.48-0.76) |  |  |  |  |  |  |  |
|  | rs121909567*CAD | 0.037 | 0.86 | (-0.92-1.1) |  |  |  |  |  |  |  |
|  | rs121909567*Migraine | 0.016 | 0.92 | (-0.56-0.62) |  |  |  |  |  |  |  |
|  | rs121909567*Depression | 0.005 | 0.99 | (-1.86-1.89) |  |  |  |  |  |  |  |
|  | rs121909567*Obesity | 0.028 | 0.88 | (-0.16-0.18) |  |  |  |  |  |  |  |
|  | rs121909567* Smoking | -0.002 | 0.99 | (-0.311-0.31) |  |  |  |  |  |  |  |
|  | rs121909567*LDL-C | -0.008 | 0.94 | (-0.0412-0.379) | -0.010 | 0.003(>0.05) |  |  |  |  |  |
|  | rs121909567*TC | 0.015 | 0.89 | (-0.342-0.392) |  |  |  |  |  |  |  |
|  | rs121909567*HDL-C | 0.168 | 0.29 | (-0.099-0.327) | 0.027 | 0.054(0.03) |  |  |  |  |  |
|  | rs121909567*TG | 0.005 | 0.97 | (-0.203-0.209) | 0.025 | 0.053(0.034) |  |  |  |  |  |
| F2 | rs1799963*DM | 0.001 | 0.99 | (-0.68-0.67) | 0.014 | 0.001(>0.05) |  |  |  | 0.049 | 0.001(>0.05) |
|  | rs1799963*Cancer |  |  |  |  |  |  |  |  |  |  |
|  | rs1799963*CKD |  |  |  |  |  |  |  |  |  |  |
|  | rs1799963*CAD | 0.038 | 0.71 | (-0.39-0.57) |  |  |  |  |  |  |  |
|  | rs1799963*Migraine | 0.016 | 0.83 | (-0.42-0.53) |  |  | 0.018 | 0.78 | (-0.19-0.25) |  |  |
|  | rs1799963*Depression | 0.008 | 0.94 | (-0.46-0.5) |  |  | 0.022 | 0.69 | (-0.21-0.32) |  |  |
|  | rs1799963*Obesity |  |  |  |  |  | 0.002 | 0.9 | (-0.13-0.14) |  |  |
|  | rs1799963* Smoking | -0.002 | 0.99 | (-0.48-0.47) |  |  | 0.008 | 0.99 | (-0.09-0.09) |  |  |
|  | rs1799963*LDL-C | - | - | - | -0.007 | 0.003(>0.05) | 0.011 | 0.94 | (-0.246-0.264) | -0.011 | 0.002(>0.05) |
|  | rs1799963* TC | 0.011 | 0.86 | (-0.273-0.324) |  |  | 0.000 | 0.998 | (-0.261-0.261) |  |  |
|  | rs1799963* HDL-C | 0.091 | 0.72 | (-0.310-0.446) | 0.021 | 0.049(0.055) | -0.091 | 0.55 | (-0.154-0.083) | 0.058 | 0.084(<0.001) |
|  | rs1799963* TG | -0.066 | 0.69 | (-0.414-0.503) | 0.023 | 0.050(0.048) | 0.030 | 0.85 | (-0.108-0.131) | 0.053 | 0.079(0.001) |
| F11 | rs2036914*DM | -0.198 | 0.05 | (-0.15-0.001) | 0.054 | 0.047(0.013) | -0.023 | 0.79 | (-0.08-0.06) | 0.082 | 0.042(0.02) |
|  | rs2036914*Cancer | -0.072 | 0.49 | (-0.27 - 0.13) |  |  | 0.024 | 0.74 | (-0.14-0.2) |  |  |
|  | rs2036914*CKD | -0.056 | 0.51 | (-0.18-0.09) |  |  | -0.013 | 0.95 | (-0.31-0.29) |  |  |
|  | **rs2036914*CAD** | **0.28** | **0.001** | **0.07-0.27** |  |  | **0.423** | **0.001** | **0.07-0.27** |  |  |
|  | rs2036914*Migraine | 0.05 | 0.57 | (-0.04-0.08) |  |  | 0.141 | 0.12 | (-0.09-0.01) |  |  |
|  | **rs2036914*Depression** | **0.819** | **0.02** | **0.02-0.18** |  |  | 0.343 | 0.33 | (-0.03-0.9) |  |  |
|  | rs2036914*Obesity | 0.115 | 0.44 | (-0.02-0.04) |  |  | 0.221 | 0.15 | (-0.01-0.04) |  |  |
|  | rs2036914*Smoking | 0.136 | 0.21 | (-0.02-0.08) |  |  | 0.116 | 0.22 | (-0.01-0.06) |  |  |
|  | rs2036914*LDL-C | 0.158 | 0.31 | (-0.038-0.119) | -0.008 | 0.005(>0.05) | 0.256 | 0.09 | (-0.008-0.103) | -0.004 | 0.008(>0.05) |
|  | rs2036914* TC | -0.102 | 0.52 | (-0.104-0.052) |  |  | -0.209 | 0.19 | (-0.09-0.018) |  |  |
|  | rs2036914* HDL-C | 0.169 | 0.32 | (-0.023-0.071) | 0.023 | 0.050(0.048) | -0.014 | 0.94 | (-0.035-0.033) | 0.058 | 0.084(<0.001) |
|  | rs2036914* TG | -0.071 | 0.69 | (-0.057-0.038) | 0.022 | 0.049(0.053) | -0.046 | 0.79 | (-0.038-0.029) | 0.054 | 0.080(0.001) |
| FGG | **rs2066865*DM** | 0.123 | 0.09 | (-0.014-0.18) | 0.086 | 0.074(<0.001) | **-0.19** | **0.01** | **(-0.22-(-0.03)** | 0.167 | 0.122(<0.001) |
|  | rs2066865*Cancer | -0.078 | 0.26 | (-0.25-0.07) |  |  | -0.036 | 0.54 | (-0.22-0.11) |  |  |
|  | **rs2066865*CKD** | **-0.349** | **<0.001** | **(-0.53– (-0.19)** |  |  | 0.114 | 0.11 | (-0.05-0.44) |  |  |
|  | **rs2066865*CAD** | **0.143** | **0.046** | **0.002-0.26** |  |  | **-0.329** | **<0.001** | **(-0.34-(-0.13)** |  |  |
|  | rs2066865*Migraine | -0.045 | 0.58 | (-0.08-0.44) |  |  | -0.127 | 0.05 | (-0.12-0.001) |  |  |
|  | **rs2066865*Depression** | **-0.16** | **0.046** | **(-0.17-(-0.002)** |  |  | -0.119 | 0.11 | (-0.11-0.1) |  |  |
|  | **rs2066865*Obesity** | **-0.411** | **0.003** | **(-0.082-(-0.017)** |  |  | 0.089 | 0.49 | (-0.02-0.03) |  |  |
|  | rs2066865*Smoking | -0.132 | 0.22 | (-0.098-0.023) |  |  | -0.013 | 0.85 | (-0.04-0.04) |  |  |
|  | **rs2066865*LDL-C** | **0.389** | **0.002** | **(0.048-0.218)** | 0.021 | 0.034(0.020) | 0.048 | 0.70 | (-0.058-0.086) | 0.003 | 0.016(>0.05) |
|  | **rs2066865* TC** | **-0.378** | **0.003** | **(-0.211-(-0.042)** |  |  | 0.099 | 0.45 | (-0.043-0.098) |  |  |
|  | **rs2066865* HDL-C** | **0.053** | **0.75** | **(-0.045-0.063)** | 0.025 | 0.052(0.04) | -0.264 | 0.12 | (-0.071-0.008) | 0.065 | 0.091(<0.001) |
|  | **rs2066865* TG** | **-0.007** | **0.96** | **(-0.055-0.052)** | 0.026 | 0.053(0.031) | 0.005 | 0.97 | (-0.039-0.041) | 0.055 | 0.081(<0.001) |
| F5 | rs6025*DM | 0.056 | 0.39 | (-0.07-0.18) | 0.096 | 0.083(<0.001) | 0.066 | 0.44 | (-0.15-0.33) | 0.097 | 0.048(0.002) |
|  | rs6025*Cancer | 0.076 | 0.21 | (-0.08-0.38) |  |  |  |  |  |  |  |
|  | **rs6025*CKD** | **-0.277** | **<0.001** | **(-1.29-(-0.59)** |  |  |  |  |  |  |  |
|  | rs6025*CAD | -0.044 | 0.51 | (-0.199-0.1) |  |  | 0.106 | 0.22 | (-0.11-0.47) |  |  |
|  | rs6025*Migraine | -0.032 | 0.58 | (-0.15-0.08) |  |  | -0.085 | 0.17 | (-0.23-0.04) |  |  |
|  | rs6025*Depression | 0.091 | 0.13 | (-0.046-0.36) |  |  | -0.075 | 0.25 | (-0.21-0.05) |  |  |
|  | rs6025*Obesity | -0.002 | 0.99 | (-0.06-0.06) |  |  | 0.145 | 0.32 | (-0.02-0.07) |  |  |
|  | **rs6025*Smoking** | -0.014 | 0.86 | (-0.09-0.08) |  |  | **0.172** | **0.008** | **0.02-0.16** |  |  |
|  | **rs6025*LDL-C** | -0.150 | 0.55 | (-0.189-0.10) | 0.000 | 0.012(>0.05) | **0.368** | **0.001** | **(0.078-0.312)** | 0.037 | 0.049(0.001) |
|  | **rs6025* TC** | 0.332 | 0.19 | (-0.047-0.233) |  |  | **-0.484** | **<0.001** | **-0.368-(-0.135)** |  |  |
|  | rs6025* HDL-C | 0.147 | 0.34 | (-0.044-0.129) | 0.027 | 0.055(0.03) | 0.302 | 0.05 | (0.000-0.130) | 0.071 | 0.097(<0.001) |
|  | **rs6025* TG** | -0.183 | 0.29 | (-0.132-0.041) | 0.029 | 0.056(0.022) | **0.400** | **0.007** | **(0.025-0.159)** | 0.075 | 0.100(<0.001) |
| ABO | **rs8176719*DM** | -0.039 | 0.63 | (-0.097-0.06) | **0.094** | **0.085(0.001)** | **0.194** | **0.014** | **0.02-0.15** | 0.189 | 0.131(<0.001) |
|  | **rs8176719*Cancer** | **0.37** | **<0.001** | **0.17-0.06** |  |  | -0.042 | 0.6 | (-0.1-0.06) |  |  |
|  | **rs8176719*CKD** | **-0.334** | **<0.001** | **(-0.5-(-0.19)** |  |  | -0.237 | 0.12 | (-0.410-0.05) |  |  |
|  | **rs8176719*CAD** | 0.088 | 0.31 | (-0.061-0.19) |  |  | **0.197** | **0.009** | **0.03-0.18** |  |  |
|  | **rs8176719*Migraine** | -0.092 | 0.26 | (-0.1-0.03) |  |  | **0.287** | **0.001** | **0.04-0.15** |  |  |
|  | **rs8176719*Depression** | 0.011 | 0.9 | (-0.08-0.09) |  |  | **0.342** | **<0.001** | **0.06-0.19** |  |  |
|  | rs8176719*Obesity | 0.064 | 0.66 | (-0.02-0.04) |  |  | -0.139 | 0.3 | (-0.03-0.01) |  |  |
|  | rs8176719*Smoking | 0.114 | 0.28 | (-0.02-0.08) |  |  | -0.007 | 0.93 | (-0.04-0.03) |  |  |
|  | rs8176719*LDL-C | -0.760 | 0.45 | (-0.105-0.046) | -0.001 | 0.012(>0.05) | -0.005 | 0.97 | (-0.051-0.049) | 0.006 | 0.018(>0.05) |
|  | rs8176719* TC | 1.529 | 0.13 | (-0.017-0.136) |  |  | -0.007 | 0.96 | (-0.051-0.049) |  |  |
|  | rs8176719* HDL-C | -0.041 | 0.81 | (-0.054-0.042) | 0.021 | 0.048(0.060) | 0.016 | 0.93 | (-0.031-0.034) | 0.071 | 0.096(<0.001) |
|  | rs8176719* TG | 0.054 | 0.77 | (-0.041-0.055) | 0.022 | 0.049(0.051) | 0.121 | 0.72 | (-0.02-0.044) | 0.068 | 0.094(<0.001) |

**β** =Standardized regression coefficient**, ADr**^2^(Adjusted R square) = Variation in the outcome due to the set of predictors in the model, r^2^ Change= Impact of the GxE on the VTE risks as a result of multiplicative interaction between VTE environmental/personal risk factors with that of genetics. ‡ the general Hungarian population genotypes data did not have a risk allele for this particular SNP, so GxE for this SNP was computed only for Roma population. Significance differences are highlighted in bold.
